# Comparison of Results in ACL Reconstruction in Women under 30 Years Old at a Minimum of 2 Years’ Follow-Up between a Bone–Tendon–Bone (BTB) Technique with the Patellar Tendon and a Hamstring Technique Combined with Anterolateral Ligament Reconstruction

**DOI:** 10.3390/jcm13206067

**Published:** 2024-10-11

**Authors:** Elio Disegni, Nicolas Pujol, Romain Letartre

**Affiliations:** 1 Ramsay Général de Santé, Hôpital Privé La Louvière, F-59800 Lille, France; 2 Versailles Arthroscopie Orthopédie, Hôpital André Mignot, F-78150 Le Chesnay, France

**Keywords:** arthroscopy, knee, ligament reconstruction, women

## Abstract

**Background**: Anterior cruciate ligament (ACL) rupture is a frequent injury among athletes, particularly women. Various techniques have shown effectiveness, but their impact on laxity and clinical outcomes varies. This study aims to compare the rupture rates of patellar tendon (PT) reconstruction versus hamstring reconstruction (HR) combined with anterolateral ligament reconstruction (ALLR) in young women. The secondary objectives include comparing functional ACL-RSI and subjective IKDC scores, as well as the Tegner scale, between these two groups. The hypothesis is that adding ALLR to HR will result in rupture rates and functional scores similar to those of PT reconstruction. **Methods**: Between 2015 and 2019, 96 patients were treated at two facilities, with 70 having an average follow-up of 44 ± 14.5 months: 35 underwent PT reconstruction, and 35 had HR combined with ALLR. Patients were re-evaluated remotely after at least 2 years using a standardised questionnaire and assessing subjective IKDC, ACL-RSI, and Tegner scores. **Results**: Rupture rates were 5.7% in both groups. The mean subjective IKDC score was 81% for the HR + ALLR group versus 80.8% for the PT group (*p* = 0.09). The mean ACL-RSI score was 66% for HR + ALLR versus 68% for PT (*p* = 0.78). The HR + ALLR group lost an average of 0.4 points on the Tegner scale postoperatively, while the PT group lost an average of 0.77 points (*p* = 0.09). **Conclusions**: Hamstring surgery combined with anterolateral surgery provides subjective results, as assessed by patients using subjective scales and questionnaires, that are as good as those obtained with PT surgery in young women. Notwithstanding, the results are not corroborated by clinical or radiological examination.

## 1. Introduction

An anterior cruciate ligament (ACL) rupture is one of the most common knee injuries [1]. It temporarily prevents sports activities, limits abilities when sport is resumed, and increases the risk of further knee injury when sports activities are resumed [2]. This injury also leads to an earlier onset of osteoarthritis of the knee [3]. Several types of management have been described and validated, ranging from orthopaedic to surgical treatment [4,5,6]. As far as surgical management is concerned, the bone–tendon–bone (BTB) technique with the patellar tendon (PT), using patellar tendon with a patellar and tibial bone rod as a graft, has long been the reference technique [7,8]. However, complications due to the harvesting site (anterior pain, risk of patellar fracture), as well as equivalence studies [3,7,9,10,11], have motivated the use of other techniques. Techniques using the hamstring semitendinosus and gracilis tendons (STG) or the semitendinosus alone (ST3 or ST4) have shown good clinical results [12,13,14] and are widely used today. These techniques have shown good results in terms of anteroposterior stability of the knee, but some patients continue to experience rotational instability, which is explained by a concomitant injury to the anterolateral ligament [15]. Anterolateral ligament reconstruction techniques were therefore developed and Sonnery-Cottet et al. showed a reduction in repeat ruptures [16] in these patients compared to treatment with hamstring grafts alone, as well as an improvement in rotational stability [17,18,19,20]. Women are more frequently affected by ACL tears than men, especially young women playing pivoting contact sports such as basketball or soccer [1,17,18,21]. This risk varies over the menstrual cycle and is increased in the pre-ovulatory phase [22]. When comparing female and male athletes, physiological studies show significantly higher tibial translation in women, weaker hamstrings, and a more frequent valgus morphotype. Each of these factors contributes to the increase in stress on the ACL [15,23,24], but without any clinical impact. In contrast to PT reconstruction, hamstring reconstruction has been shown to increase laxity in women compared with men [13,25], without any clinical consequences. Some authors have also found an increased postoperative laxity and repeat rupture rate after hamstring surgery compared to PT reconstruction in a population of female athletes [9]. To our knowledge, no study has compared PT reconstruction to the hamstring technique with anterolateral ligament reconstruction in the young female population. The main objective of our study is to compare the rupture rate of patellar tendon reconstruction with the rupture rate of hamstring reconstruction combined with anterolateral ligament reconstruction. The secondary objectives are to compare the postoperative functional ACL-RSI and subjective IKDC scores and the Tegner scale in these two groups. The hypothesis of our study is that adding anterolateral ligament reconstruction when performing hamstring reconstruction results in the same repeat rupture rate and functional scores as PT reconstruction.

## 2. Materials and Methods

We conducted a retrospective, multicentre study at the Centre Hospitalier de Versailles and the Hôpital Privé de la Louvière. Patients treated between 2015 and 2019 were included. The inclusion criteria were women who underwent ACL reconstruction, aged between 15 and 30 years at the time of surgery, using one of the following techniques: HR + ALLR or the patellar tendon technique. Only patients aged 17 years and older at follow-up were included. During the COVID 19 period, the patients did not have in-person consultations. A telephone follow-up was carried out during which we assessed the subjective IKDC score [26] (Supplementary Materials S1 and S2) and performed the ACL-RSI test [27] (Supplementary Materials S3) and the Tegner activity scale [28] (Supplementary Materials S4).

The Tegner score was assessed once for the period before the ACL rupture and again for the period corresponding to the current follow-up. A Tegner score differential was then calculated to best assess the patients’ return to sport. All morphological data were collected by telephone.

The HR + ALLR group included a total of 42 patients; 7 patients had changed telephone numbers or were unreachable. The PT group included 54 patients; 15 patients had changed telephone numbers and 4 did not answer all questionnaires. Our study included 35 patients in the PT group and 35 patients in the HR + ALLR group. The mean follow-up time was 44 ± 14.5 months for both groups (*p* = 0.25). The mean age of the patients was 24 years with no difference between the two groups (*p* = 0.08). There was no significant difference in height (*p* = 0.17), weight (*p* = 0.65), or body mass index (BMI) (*p* = 0.83) between the two groups (Table 1). The preoperative Tegner score of the HR + ALLR group (Tegner = 7.86) was significantly higher than that of the PT group (Tegner = 6.34) (*p* < 0.001).

At Versailles Hospital, several senior surgeons were involved, all of whom used the PT technique: the femoral tunnel is drilled inside-out through an independent anteromedial arthroscopic incision with tibial and femoral fixation using interference screws [29]. At the Hôpital Privé de la Louvière, there was a single senior surgeon performing the HR + ALLR technique: the femoral tunnel is drilled outside-in, and the ST3 is left pedicled on its distal enthesis. The single-strand gracilis is attached to the ST3 in the tibial tunnel, in the intercondylar notch, and in the femoral tunnel. It exits the lateral femoral condyle 5 mm posteriorly and 5 mm proximally to the lateral epicondyle. It is then looped to form a double gracilis strand and passed under the fascia lata and stapled to the tibia behind Gerdy’s tubercle. The ST3 + single-strand gracilis is attached with an interference screw in the tibial tunnel and in the femoral tunnel.

Statistical analysis was performed using EasyMedStat software (version 3.7; www.easymedstat.com, accessed on 20 June 2021). Comparisons of mean follow-up times, ages, and years of inclusion were calculated using Student’s unpaired *t*-test. Comparisons of the mean clinical scores were calculated using a Mann–Whitney test. Comparisons of the mean morphological data were calculated with a Mann–Whitney test. Categorical variables such as the comparison of repeat ruptures were calculated with a Fisher test.

## 3. Results

### 3.1. Results in the PT Group

Two patients (5.7%) presented with a reconstruction tear at 3 and 4 years postoperatively and were not reoperated on. The mean subjective IKDC score was 81.32% ± 11.5 with a maximum score of 100% and a median of 82.76. The mean ACL-RSI score was 67.83% ± 23.2 with a maximum score of 100% and a median of 75.83. The mean Tegner scale score before the injury was 6.34 ± 0.39 [4,5,6,7,8,9] with a maximum score of 10 and a median of 6. The mean Tegner score for the current follow-up period was 5.57 ± 1.17 [3,4,5,6,7] and the median was 6. The Tegner differential was a loss of 0.77 points.

### 3.2. Results in the HR + ALLR Group

Two patients (5.7%) presented with a reconstruction tear at 2.5 years and 4 years postoperatively, one of which was reoperated on. The mean subjective IKDC score was 80.89% ± 14.5 with a maximum score of 100% and a median of 83.90. The mean ACL-RSI score was 66.26% ± 22.9 with a maximum score of 100% and a median of 67.50. The mean Tegner scale score before the injury was 7.86 ± 1.5 [4,5,6,7,8,9,10] with a maximum score of 10 and a median of 9. The mean Tegner score for the current follow-up period was 7.46 ± 1.8 [4,5,6,7,8,9,10] and the median was 7. The Tegner differential was a loss of 0.4 points. One patient had a postoperative Tegner score 1 point higher than her preoperative score.

### 3.3. Comparison of the Two Groups

**Figure 1:**
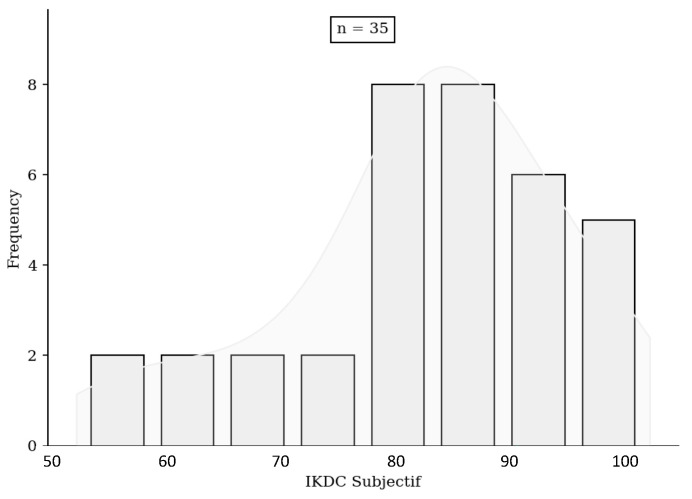
IKDC score comparison.

**Figure 2:**
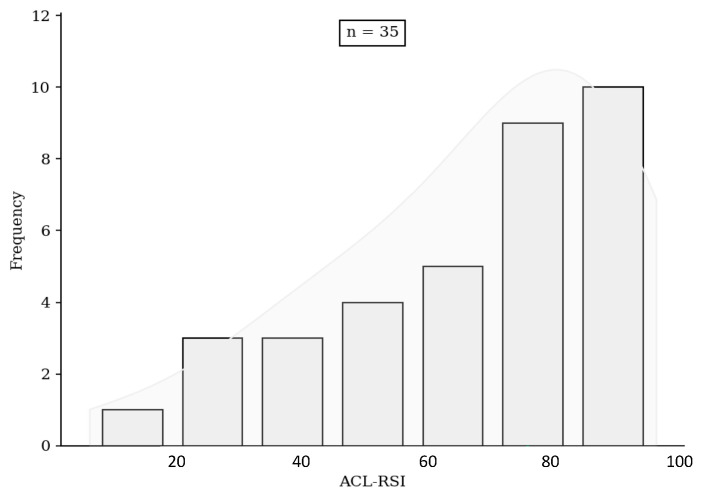
ACL-RSI score comparison.

**Figure 3:**
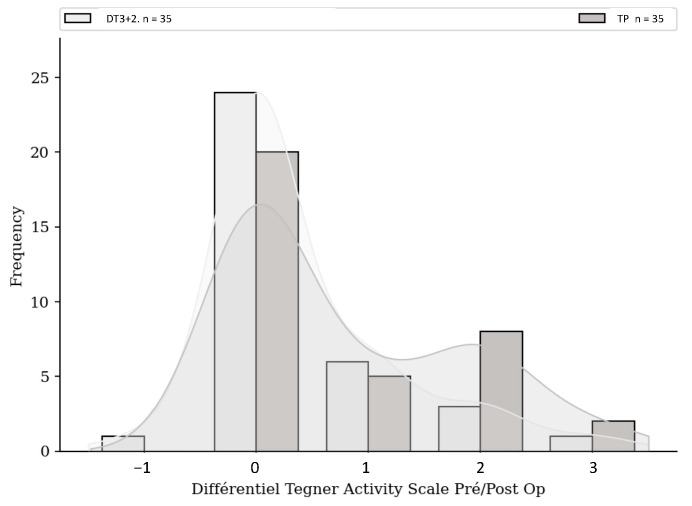
Tegner differential comparison.

**Figure 4:**
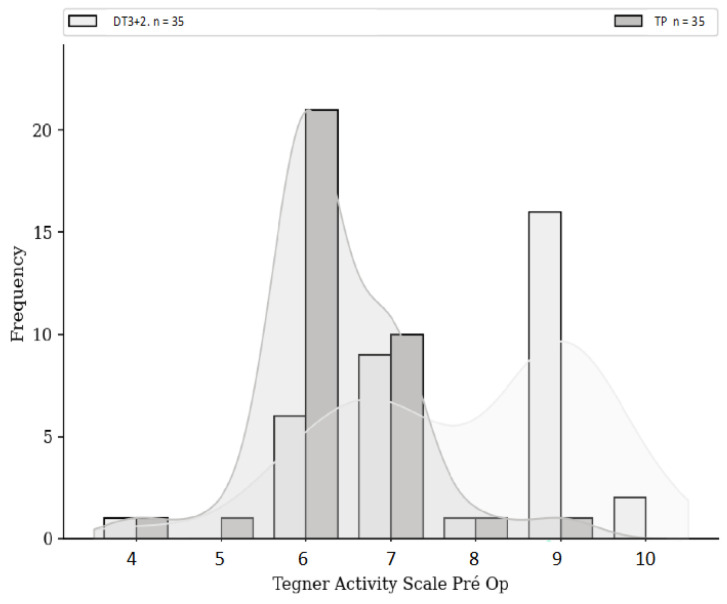
Preoperative Tegner score comparison.

**Figure 5:**
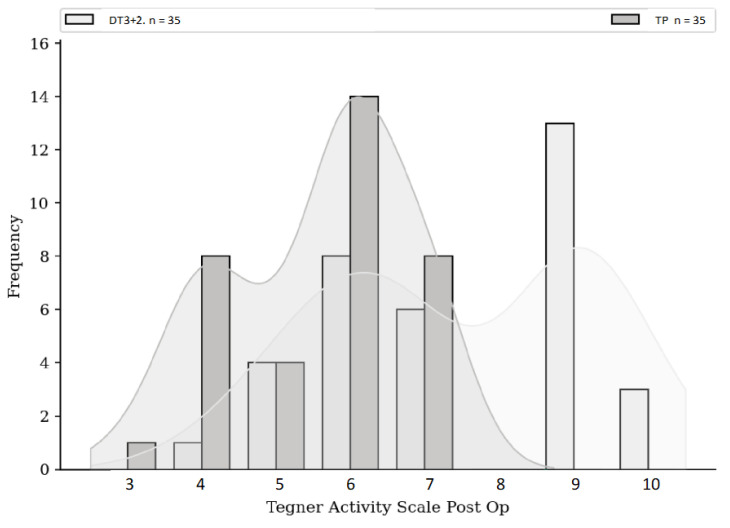
Postoperative Tegner score comparison.

No significant difference was found either in terms of risk of rupture, subjective IKDC score (Figure 1), ACL-RSI score (Figure 2), or Tegner score differential (Figure 3). Patients in the HR + ALLR group had significantly higher preoperative (Figure 4) and postoperative (Figure 5) Tegner scores than patients in the PT group (*p* < 0.001). The statistical values are summarised in Table 2.

## 4. Discussion

Multiple studies have compared complications in patients having hamstring and PT surgeries, in particular the rate of repeat rupture. Samuelsen performed a meta-analysis of more than 47,000 patients and found a small decrease in the risk of repeat rupture in patients having hamstring surgery [14]. However, other studies [30], some of which had a long follow-up [5], show no significant difference in the risk of repeat rupture between the two techniques. In other series, there was a tendency for the hamstring technique without anterolateral ligament reconstruction to result in more ruptures than the PT technique [31], whereas in our study, adding anterolateral ligament reconstruction at the time of the hamstring procedure resulted in functional results and a repeat rupture rate identical to the PT procedure in young women. Also, the subjective IKDC and ACL-RSI scores are similar in the two groups, which is not the case for hamstring reconstruction without anterolateral ligament reconstruction [13,32]. When looking at the literature, similar postoperative clinical outcomes are found in men and women after ACL reconstruction, although men have more concomitant meniscal injuries [21]. Similarly, clinical outcomes between men and women do not differ, regardless of graft type [7]. Ryan, in his meta-analysis of 13 studies in the literature, found no postoperative difference between men and women in terms of risk of repeat rupture or contralateral rupture [33]. The patients in the HR + ALLR group had a higher preoperative and postoperative athletic level than patients in the PT group. However, when we look at the Tegner differential, the difference in favour of a better return to sport in the HR + ALLR group does not seem significant. Yet this does show an improvement compared to studies comparing the PT technique with the hamstring technique without anterolateral ligament reconstruction [9]. Our study has several limitations: the main limitation is the absence of any clinical examination in the comparison of the patient groups. Furthermore, as follow-up data were collected by telephone, this can introduce recall bias and may not show subtle differences in clinical outcomes. The various tests used are relevant in gauging what the patient feels and to assess her return to sport, but they do not allow us to study laxity and pivot shift. The presence of a reconstruction tear is only described according to what the patient feels, but it can be assumed that if each patient benefited from a thorough clinical examination and/or an imaging examination such as MRI, several false negatives would be discovered [34]. Some authors have found a significant decrease in the pivot shift test when an extra-articular tenodesis is added [35]. Furthermore, we did not take into account the presence or absence of a meniscal lesion during ligament reconstruction in our patients, or its surgical management, yet we know that the presence of an unmanaged meniscal injury influences the results of ligament reconstruction [36,37]. In addition, the interval between the initial injury and the reconstruction surgery was not taken into account, which may include a bias by giving poorer results to patients who had a longer interval before surgery [38]. The number of patients surveyed may be a limiting factor: the Tegner score differential appears to be greater in the PT group, but it is not significant. A larger population would increase the power and perhaps show a significant difference. Lastly, the HR + ALLR population has a significantly higher athletic level than patients in the PT group, which may lead to a recruitment bias and distort the comparability of the groups studied. This recruitment bias is also found when comparing our Tegner score to that found in other articles in the literature [39].

## 5. Conclusions

Hamstring surgery combined with anterolateral surgery provides subjective results, as assessed by patients using subjective scales and questionnaires, that are as good as those obtained with PT surgery in young women. Further studies, with specific elements of the clinical examination of the knee as primary and secondary endpoints, should be performed to better compare these two techniques.

## Figures and Tables

**Table 1:** Comparison of morphological features.

Variables	PT	HR + ALLR	*p*-Value
	(n = 35)	(n = 35)	
Height (cm)			
Mean	165.34	167.37	*p* = 0.17
Standard Deviation	6.79	5.45	
Weight (kg)			
Mean	61.43	62.54	*p* = 0.65
Standard Deviation	11.69	9.01	
BMI (Body Mass Index)			
Mean	22.39	22.31	*p* = 0.83
Standard Deviation	4.05	2.77	
Preoperative Tegner			
Mean	6.34	7.86	*p* < 0.001
Standard Deviation	0.84	1.46	

**Table 2:** Statistical results.

Variables	PT	HR + ALLR	*p*-Value
	(n = 35)	(n = 35)	
Reconstruction Tear	5.70%	5.70%	*p* = 1
Subjective IKDC			
Mean	81.31	80.89	*p* = 0.9
Standard Deviation	11.54	14.49	
ACL-RSI			
Mean	66.26	66.26	*p* = 0.78
Standard Deviation	23.23	22.89	
Postoperative Tegner			
Mean	5.57	7.46	*p* < 0.001
Standard Deviation	1.17	1.75	
Tegner Differential	0.77	0.4	*p* = 0.09

## Data Availability

The original contributions presented in the study are included in the article/Supplementary Material, further inquiries can be directed to the corresponding author.

## Supplementary Materials

The following supporting information can be downloaded at: https://www.mdpi.com/article/10.3390/jcm13206067/s1.

## References

[1] Acevedo R.J., Rivera-Vega A., Miranda G., Micheo W. (2014). Anterior cruciate ligament injury: Identification of risk factors and prevention strategies. Curr. Sports Med. Rep..

[2] Nagelli C.V., Hewett T.E. (2017). Should Return to Sport be Delayed Until 2 Years after Anterior Cruciate Ligament Reconstruction? Biological and Functional Considerations. Sports Med..

[3] Kautzner J., Kos P., Hanus M., Trc T., Havlas V. (2015). A comparison of ACL reconstruction using patellar tendon versus hamstring autograft in female patients: A prospective randomised study. Int. Orthop..

[4] Smith T.O., Postle K., Penny F., McNamara I., Mann C.J.V. (2014). Is reconstruction the best management strategy for anterior cruciate ligament rupture? A systematic review and meta-analysis comparing anterior cruciate ligament reconstruction versus non-operative treatment. Knee.

[5] Sajovic M., Strahovnik A., Dernovsek M.Z., Skaza K. (2011). Quality of life and clinical outcome comparison of semitendinosus and gracilis tendon versus patellar tendon autografts for anterior cruciate ligament reconstruction: An 11-year follow-up of a randomized controlled trial. Am. J. Sports Med..

[6] Hofmeister E.P., Gillingham B.L., Bathgate M.B., Mills W.J. (2001). Results of anterior cruciate ligament reconstruction in the adolescent female. J. Pediatr. Orthop..

[7] Svensson M., Sernert N., Ejerhed L., Karlsson J., Kartus J.T. (2006). A prospective comparison of bone-patellar tendon-bone and hamstring grafts for anterior cruciate ligament reconstruction in female patients. Knee Surg. Sports Traumatol. Arthrosc..

[8] Gifstad T., Foss O.A., Engebretsen L., Lind M., Forssblad M., Albrektsen G., Drogset J.O. (2014). Lower risk of revision with patellar tendon autografts compared with hamstring autografts: A registry study based on 45,998 primary ACL reconstructions in Scandinavia. Am. J. Sports Med..

[9] Barrett G.R., Noojin F.K., Hartzog C.W., Nash C.R. (2002). Reconstruction of the anterior cruciate ligament in females: A comparison of hamstring versus patellar tendon autograft. Arthroscopy.

[10] Thaunat M., Fayard J.M., Sonnery-Cottet B. (2019). Hamstring tendons or bone-patellar tendon-bone graft for anterior cruciate ligament reconstruction?. Orthop. Traumatol. Surg. Res..

[11] Hardy A., Casabianca L., Andrieu K., Baverel L., Noailles T. (2017). Junior French Arthroscopy Society. Complications following harvesting of patellar tendon or hamstring tendon grafts for anterior cruciate ligament reconstruction: Systematic review of literature. Orthop. Traumatol. Surg. Res..

[12] Wipfler B., Donner S., Zechmann C.M., Springer J., Siebold R., Paessler H.H. (2011). Anterior cruciate ligament reconstruction using patellar tendon versus hamstring tendon: A prospective comparative study with 9-year follow-up. Arthroscopy.

[13] Stevanović V., Blagojević Z., Basarević Z., Tomić S., Crnobarić A. (2006). Anterior cruciate ligament reconstruction: Comparison of male and female athletes using the patellar tendon and hamstring autografts. Acta Chir. Iugosl..

[14] Samuelsen B.T., Webster K.E., Johnson N.R., Hewett T.E., Krych A.J. (2017). Hamstring Autograft versus Patellar Tendon Autograft for ACL Reconstruction: Is There a Difference in Graft Failure Rate? A Meta-analysis of 47,613 Patients. Clin. Orthop. Relat. Res..

[15] Getgood A., Brown C., Lording T., Amis A., Claes S., Geeslin A., Musahl V. (2019). The anterolateral complex of the knee: Results from the International ALC Consensus Group Meeting. Knee Surg. Sports Traumatol. Arthrosc..

[16] Sonnery-Cottet B., Barbosa N.C., Vieira T.D., Saithna A. (2018). Clinical outcomes of extra-articular tenodesis/anterolateral reconstruction in the ACL injured knee. Knee Surg. Sports Traumatol. Arthrosc..

[17] Guzzini M., Mazza D., Fabbri M., Lanzetti R., Redler A., Iorio C., Monaco E., Ferretti A. (2016). Extra-articular tenodesis combined with an anterior cruciate ligament reconstruction in acute anterior cruciate ligament tear in elite female football players. Int. Orthop..

[18] Vadalà A.P., Iorio R., De Carli A., Bonifazi A., Iorio C., Gatti A., Rossi C., Ferretti A. (2013). An extra-articular procedure improves the clinical outcome in anterior cruciate ligament reconstruction with hamstrings in female athletes. Int. Orthop..

[19] Slette E.L., Mikula J.D., Schon J.M., Marchetti D.C., Kheir M.M., Turnbull T.L., LaPrade R.F. (2016). Biomechanical Results of Lateral Extra-articular Tenodesis Procedures of the Knee: A Systematic Review. Arthroscopy.

[20] Lutz C. (2018). Role of anterolateral reconstruction in patients undergoing anterior cruciate ligament reconstruction. Orthop. Traumatol. Surg. Res..

[21] Wiger P., Brandsson S., Kartus J., Eriksson B.I., Karlsson J. (1999). A comparison of results after arthroscopic anterior cruciate ligament reconstruction in female and male competitive athletes. A two- to five-year follow-up of 429 patients. Scand. J. Med. Sci. Sports.

[22] Lefevre N., Bohu Y., Klouche S., Lecocq J., Herman S. (2013). Anterior cruciate ligament tear during the menstrual cycle in female recreational skiers. Orthop. Traumatol. Surg. Res..

[23] Huston L.J., Wojtys E.M. (1996). Neuromuscular performance characteristics in elite female athletes. Am. J. Sports Med..

[24] Medrano D., Smith D. (2003). A comparison of knee joint laxity among male and female collegiate soccer players and non-athletes. Sports Biomech..

[25] Paterno M.V., Weed A.M., Hewett T.E. (2012). A between sex comparison of anterior-posterior knee laxity after anterior cruciate ligament reconstruction with patellar tendon or hamstrings autograft: A systematic review. Sports Med..

[26] Grevnerts H., Terwee C., Kvist J. (2015). The measurement properties of the IKDC-subjective knee form. Knee Surg. Sports Traumatol. Arthrosc..

[27] Bohu Y., Klouche S., Lefevre N., Webster K., Herman S. (2015). Translation, cross-cultural adaptation and validation of the French version of the Anterior Cruciate Ligament-Return to Sport after Injury (ACL-RSI) scale. Knee Surg. Sports Traumatol. Arthrosc..

[28] Briggs K., Lysholm J., Tegner Y., Rodkey W., Kocher M., Steadman J. (2009). The reliability, validity, and responsiveness of the Lysholm score and Tegner activity scale for anterior cruciate ligament injuries of the knee: 25 years later. Am. J. Sports Med..

[29] Beaufils P., Gaudot F., Drain O., Boisrenoult P., Pujol N. (2011). Mini-invasive technique for bone patellar tendon bone harvesting: Its superiority in reducing anterior knee pain following ACL reconstruction. Curr. Rev. Musculoskelet. Med..

[30] Andernord D., Björnsson H., Petzold M., Eriksson B.I., Forssblad M., Karlsson J., Samuelsson K. (2014). Surgical Predictors of Early Revision Surgery After Anterior Cruciate Ligament Reconstruction: Results from the Swedish National Knee Ligament Register on 13,102 Patients. Am. J. Sports Med..

[31] Salem H.S., Varzhapetyan V., Patel N., Dodson C.C., Tjoumakaris F.P., Freedman K.B. (2019). Anterior Cruciate Ligament Reconstruction in Young Female Athletes: Patellar Versus Hamstring Tendon Autografts. Am. J. Sports Med..

[32] Beynnon B.D., Johnson R.J., Fleming B.C., Kannus P., Kaplan M., Samani J., Renström P. (2002). Anterior cruciate ligament replacement: Comparison of bone-patellar tendon-bone grafts with two-strand hamstring grafts. A prospective, randomized study. J. Bone Joint Surg. Am..

[33] Ryan J., Magnussen R.A., Cox C.L., Hurbanek J.G., Flanigan D.C., Kaeding C.C. (2014). ACL reconstruction: Do outcomes differ by sex? A systematic review. J. Bone Joint Surg. Am..

[34] Colombet P., Jenny J.Y., Menetrey J., Plaweski S., Zaffagnini S. (2012). French Arthroscopy Society (SFA). Current concept in rotational laxity control and evaluation in ACL reconstruction. Orthop. Traumatol. Surg. Res..

[35] Ventura A., Legnani C., Boisio F., Borgo E., Peretti G.M. (2021). The association of extra-articular tenodesis restores rotational stability more effectively compared to contralateral hamstring tendon autografts ACL reconstruction alone in patients undergoing ACL revision surgery. Orthop. Traumatol. Surg. Res..

[36] Kocher M.S., Steadman J.R., Briggs K., Zurakowski D., Sterett W.I., Hawkins R.J. (2002). Determinants of patient satisfaction with outcome after anterior cruciate ligament reconstruction. J. Bone Joint Surg. Am..

[37] Gonçalves H., Steltzlen C., Boisrenoult P., Beaufils P., Pujol N. (2017). High failure rate of anterior cruciate ligament reconstruction with bimeniscal repair: A case-control study. Orthop. Traumatol. Surg. Res..

[38] Newman J.T., Carry P.M., Terhune E.B., Spruiell M., Heare A., Mayo M., Vidal A.F. (2014). Delay to Reconstruction of the Adolescent Anterior Cruciate Ligament: The Socioeconomic Impact on Treatment. Orthop. J. Sports Med..

[39] Meynard P., Pelet H., Angelliaume A., Legallois Y., Lavignac P., De Bartolo R., Fabre T., Costes S. (2020). ACL reconstruction with lateral extra-articular tenodesis using a continuous graft: 10-year outcomes of 50 cases. Orthop. Traumatol. Surg. Res..

