# Supplementary material for: Comparison of Results in ACL Reconstruction in Women under 30 Years Old at a Minimum of 2 Years’ Follow-Up between a Bone–Tendon–Bone (BTB) Technique with the Patellar Tendon and a Hamstring Technique Combined with Anterolateral Ligament Reconstruction
Source: J Clin Med. 2024 Oct 11;13(20):6067. doi: 10.3390/jcm13206067 (PMC11508911; doi:10.3390/jcm13206067)
Supplement: Supplementary file 1 [file jcm-13-06067-s001.zip › Supplementary S1.pdf]

Supplementary S1: Subjective IKDC Score

1999 SUBJECTIVE KNEE EVALUATION FORM

Your full name ________________________________

Today's date ____/____/____

day month year

Date of injury ____/____/____

day month year

SYMPTOMS

1. What is the highest level of activity that you can perform without significant knee pain?

   ☐ Very strenuous activities like jumping or pivoting as in basketball or soccer
   ☐ Strenuous activities like heavy physical work, skiing or tennis
   ☐ Moderate activities like moderate physical work, running or jogging
   ☐ Light activities like walking, housework or yard work
   ☐ Unable to perform any of the above activities due to knee pain

2. During the past 4 weeks or since your injury, how often have you had knee pain?

	0	1	2	3	4	5	6	7	8	9	10
Never	☐	☐	☐	☐	☐	☐	☐	☐	☐	☐	☐ Constant

3. If you have pain; how severe is it?

	0	1	2	3	4	5	6	7	8	9	10
No pain	☐	☐	☐	☐	☐	☐	☐	☐	☐	☐	☐ Worst pain imaginable

4. During the past 4 weeks, or since your injury, how stiff or swollen was your knee?

   ☐ Not at all
   ☐ Mildly
   ☐ Moderately
   ☐ Very
   ☐ Extremely

5. What is the highest level of activity you can perform without significant swelling in your knee?

   ☐ Very strenuous activities like jumping or pivoting as in basketball or soccer
   ☐ Strenuous activities like heavy physical work, skiing or tennis
   ☐ Moderate activities like moderate physical work, running or jogging
   ☐ Light activities like walking, housework or yard work
   ☐ Unable to perform any of the above activities due to knee pain

6. During the past 4 weeks, or since your injury, did your knee lock or catch?

   ☐ Yes ☐ No

7. What is the highest level of activity you can perform without significant giving way in your knee?

   ☐ Very strenuous activities like jumping or pivoting as in basketball or soccer
   ☐ Strenuous activities like heavy physical work, skiing or tennis
   ☐ Moderate activities like moderate physical work, running or jogging
   ☐ Light activities like walking, housework or yard work
   ☐ Unable to perform any of the above activities due to knee pain
