# Supplementary material for: Comparison of Results in ACL Reconstruction in Women under 30 Years Old at a Minimum of 2 Years’ Follow-Up between a Bone–Tendon–Bone (BTB) Technique with the Patellar Tendon and a Hamstring Technique Combined with Anterolateral Ligament Reconstruction
Source: J Clin Med. 2024 Oct 11;13(20):6067. doi: 10.3390/jcm13206067 (PMC11508911; doi:10.3390/jcm13206067)
Supplement: Supplementary file 1 [file jcm-13-06067-s001.zip › Supplementary S2.pdf]

Supplementary S2: Subjective IKDC Score Calculation

SCORING INSTRUCTIONS FOR THE 1999 IKDC SUBJECTIVE KNEE EVALUATION FORM

The responses to each item are scored using an ordinal method such that a score of 1 is given to responses that represent the lowest level of function or highest level of symptoms.
For example, item 1, which is related to the highest level of activity without significant pain, is scored by assigning a score of 2 to the response “Light activities like walking, housework or yard work” and a score of 5 to the response “Very strenuous activities like jumping or pivoting as in basketball or soccer”.
For item 2, which is related to the frequency of pain over the past 4 weeks, the response “Constant” is assigned a score of 1 and “Never” is assigned a score of 11.
The functional impact is assessed by the highest level of activity at which the patient thinks they are asymptomatic. The IKDC Subjective Knee Evaluation Form is scored by summing the scores for the individual items on a scale that ranges from 0 to 100. This IKDC score is calculated as follows:

1. A specific score is assigned to each item so that the lowest score represents the lowest level of activity or the highest level of symptoms
2. The “raw score”, i.e. the sum of the different item scores, is calculated
3. The “raw score” is transformed to an IKDC score on a scale from 0 to 100 as follows:

$$\text{IKDC score} = \frac{\text{Raw score} - \text{Minimum score}}{\text{Difference between extreme scores}}$$

The lowest or minimum score is 18 and the highest score is 101.
The difference between extreme scores is 105-18 i.e. 87. In this specific case, if the sum of the scores of the 18 items equals 60 (=raw score), the IKDC score will be as follows:

$$\text{IKDC score} = \frac{60 - 18}{87} \times 100 = 50.6$$

The IKDC score obtained corresponds to a functional evaluation so that its maximum value represents the highest level of activity or lowest level of symptoms present. An IKDC score of 100 equates to an unlimited level of daily or sports activities without any symptoms.

The IKDC Subjective Knee Form score can be calculated when there are responses to at least 90% of the items (i.e. when responses have been provided for at least 16 out of 18 items).
In such cases, when information is missing, the raw score is calculated by assigning an average score to unanswered items. Once the raw score is calculated in this way, the IKDC score is calculated as described above.

Do not include the “Function Prior to Knee Injury” item in the subjective knee evaluation score.
