# Supplementary material for: Comparison of Results in ACL Reconstruction in Women under 30 Years Old at a Minimum of 2 Years’ Follow-Up between a Bone–Tendon–Bone (BTB) Technique with the Patellar Tendon and a Hamstring Technique Combined with Anterolateral Ligament Reconstruction
Source: J Clin Med. 2024 Oct 11;13(20):6067. doi: 10.3390/jcm13206067 (PMC11508911; doi:10.3390/jcm13206067)
Supplement: Supplementary file 1 [file jcm-13-06067-s001.zip › Supplementary S3.pdf]

Supplementary S3: ACL-RSI Scale

ACL-RSI scale
French adaptation

Patient: ………………………………………………………… Date of birth ___/___/___

Side concerned: Left ☐ Right ☐

Date of evaluation ___/___/___ Date of surgery ___/___/___

Instructions:

Please answer the following questions about the main sport you played before the injury. For each question, tick the box between the two extremes to show what best describes your current knee situation.

1. Do you think that you can play your sport at your previous level?

Not sure	0	1	2	3	4	5	6	7	8	9	10	Totally
at all	☐	☐	☐	☐	☐	☐	☐	☐	☐	☐	☐	sure

2. Do you think you could re-injure your knee by playing your sport again?

Extremely	0	1	2	3	4	5	6	7	8	9	10	Not likely
likely	☐	☐	☐	☐	☐	☐	☐	☐	☐	☐	☐	at all

3. Are you worried about playing your sport again?

Extremely	0	1	2	3	4	5	6	7	8	9	10	Not worried
worried	☐	☐	☐	☐	☐	☐	☐	☐	☐	☐	☐	at all

4. Are you confident that your knee will not give way by playing your sport?

Not sure	0	1	2	3	4	5	6	7	8	9	10	Totally
at all	☐	☐	☐	☐	☐	☐	☐	☐	☐	☐	☐	sure

5. Are you confident that you could play your sport without concern for your knee?

Not sure	0	1	2	3	4	5	6	7	8	9	10	Totally
at all	☐	☐	☐	☐	☐	☐	☐	☐	☐	☐	☐	sure

Y Bohu, S Klouche, N Lefevre, K Webster, S Herman. *Translation, cross-cultural adaptation and validation of the French version of the Anterior Cruciate Ligament-Return to Sport after Injury (ACL-RSI) scale*.
Knee Surg Sports Traumatol Arthrose 2014. DOI 10.1007/s00167-014-2942-4
