# Supplementary material for: Comparison of Results in ACL Reconstruction in Women under 30 Years Old at a Minimum of 2 Years’ Follow-Up between a Bone–Tendon–Bone (BTB) Technique with the Patellar Tendon and a Hamstring Technique Combined with Anterolateral Ligament Reconstruction
Source: J Clin Med. 2024 Oct 11;13(20):6067. doi: 10.3390/jcm13206067 (PMC11508911; doi:10.3390/jcm13206067)
Supplement: Supplementary file 1 [file jcm-13-06067-s001.zip › Supplementary S4.pdf]

Supplementary S4: Tegner Score

Patient's surname: ………………………………………………………. Date of birth: …../…../…..
First name: ……………………………………………………………….. Examination date: …../…../…..

Lysholm-Tegner Activity Scale

10	Competitive sports – soccer: national and international elite.
9	Competitive sports – soccer, lower divisions, ice hockey, gymnastics.
8	Competitive sports: squash or badminton, athletics (jumping, etc.), downhill skiing.
7	Competitive sports: tennis, athletics (running), motocross, speedway, handball, basketball. Recreational sports: soccer, ice hockey, squash, athletics (jumping), cross-country track.
6	Recreational sports: tennis and badminton, handball, basketball, downhill skiing, jogging at least five times per week.
5	Competitive sports: cycling. Recreational sports: jogging on uneven ground at least twice weekly. Heavy labour (e.g. building).
4	Recreational sports: cycling, jogging on even ground at least twice weekly. Moderately heavy labour (e.g. truck driving, heavy domestic work).
3	Competitive and recreational sports: swimming, light labour, walking in forest possible.
2	Light labour. Impossible to walk in forest.
1	Sedentary work, walking on even ground possible.
0	Sick leave or disability pension.
